# High-resolution Digital Surface Model of the 2021 eruption deposit of Cumbre Vieja volcano, La Palma, Spain

**DOI:** 10.1038/s41597-022-01551-8

**Published:** 2022-07-28

**Authors:** Riccardo Civico, Tullio Ricci, Piergiorgio Scarlato, Jacopo Taddeucci, Daniele Andronico, Elisabetta Del Bello, Luca D’Auria, Pedro A. Hernández, Nemesio M. Pérez

**Affiliations:** 1 Istituto Nazionale di Geofisica e Vulcanologia, Roma, Italy; 2 Istituto Nazionale di Geofisica e Vulcanologia, Osservatorio Etneo, Catania, Italy; 3 Instituto Volcanológico de Canarias (INVOLCAN), San Cristóbal de La Laguna, Tenerife, Canary Islands Spain; 4 Instituto Tecnológico y de Energías Renovables (ITER), Granadilla de Abona, Tenerife, Canary Islands Spain

**Keywords:** Volcanology, Natural hazards

## Abstract

Identifying accurate topographic variations associated with volcanic eruptions plays a key role in obtaining information on eruptive parameters, volcano structure, input data for volcano processes modelling, and civil protection and recovery actions. The 2021 eruption of Cumbre Vieja volcano is the largest eruptive event in the recorded history for La Palma Island. Over the course of almost 3 months, the volcano produced profound morphological changes in the landscape affecting both the natural and the anthropic environment over an area of tens of km^2^. We present the results of a UAS (Unoccupied Aircraft System) survey consisting of >12,000 photographs coupled with Structure-from-Motion photogrammetry that allowed us to produce a very-high-resolution (0.2 m/pixel) Digital Surface Model (DSM). We characterised the surface topography of the newly formed volcanic landforms and produced an elevation difference map by differencing our survey and a pre-event surface, identifying morphological changes in detail. The present DSM, the first one with such a high resolution to our knowledge, represents a relevant contribution to both the scientific community and the local authorities.

## Abstract (summary)

**Table:** 

Measurement(s)	Topographic data
Technology Type(s)	Unoccupied Aircraft System; Photogrammetry
Sample Characteristic - Location	La Palma, Canary Islands, Spain

## Background & Summary

The morphology of active volcanoes is dynamically shaped by eruptive activity and erosional processes acting at different timescales. Consequently, a precise digital elevation model is fundamental for mapping volcanic hazards, modelling volcanic processes, and complementing further analysis. Furthermore, in urbanised areas, detailed post-eruption topography is important for land recovery actions. Volcano morphologies can be quantified using different techniques^1–9^. Recently, the increased capability of UASs and their applications for aerial observation^10,11^, together with the parallel development of Structure-from-Motion (SfM) process^12^, brought important and valuable advantages compared to the classical ground-based, satellite, and crewed aircraft surveys. Nowadays, UAS-based photogrammetry is routinely applied on volcanoes to obtain very-high-resolution DSMs^13–16^.

Cumbre Vieja is the active volcanic rift on La Palma and has seen the largest number of eruptions of the Canary archipelago in historic times^17^, and its 2021 eruption was the largest eruptive event in recorded history for La Palma. The previous eruption occurred in the southern part of the island between September and November 1971. The 2021 eruption was preceded by an unrest phase characterised by increased ground deformation starting from 2009^18^, increased seismicity from 2017^19–22^, and detection of geochemical anomalies from 2010^23^. A dramatic evolution of the seismicity began on 11 September 2021 with a seismic swarm characterised by an upward migration of the hypocentres reflecting the rising of magma towards the surface.

The volcanic eruption at Cumbre Vieja started on September 19, interrupting its 50-years-long period of quiescence, and lasted until December 13 (85 days and 8 hours^24^). During this period, the volcanic activity was distributed along a fissure where a multiple-vents volcanic edifice formed (called “Volcán de Tajogaite’’). The explosive activity was characterised by alternating strombolian explosions and lava fountaining episodes, accompanied by abundant lava effusion. All such phenomena produced profound morphological changes in the landscape and severely affected settlements and industry. A total of about 12 km^2^ of territory, more than 1,600 buildings and 200 hectares of banana plantations (the island’s main economic resource after tourism), and important infrastructures (roads, powerlines, waterlines, etc.) were buried and destroyed by lava flows in their 6-km-long-path to the ocean. Here they expanded into two lava deltas, forming new land. In addition, the tephra fallout further affected the whole island and, to a smaller extent, the nearby islands of El Hierro, La Gomera and Tenerife.

Here, we present the results of a UAS survey carried out between 24 and 28 January 2022 using a DJI Phantom 4 RTK (real-time kinematic). The aerial images were georeferenced using an onboard RTK receiver capable of cm-level positioning accuracy. The dataset was then processed using Structure-from-Motion (SfM) photogrammetry and 40 Ground Control Points (GCPs) acquired between 23 and 27 January using the Differential Global Navigation Satellite System (DGNSS) positioning. This allowed us to achieve horizontal and vertical centimetre accuracy and to produce a very-high-resolution (0.2 m/pixel) Digital Surface Model (DSM) and orthophotomosaic (0.1 m/pixel), covering an area of about 17 km^2^.

Topographic change detection was obtained by differencing our survey and a pre-event (2015) 2m-pixel DTM^25^, thus identifying elevation changes at decimetre level precision. We characterised the whole topography of the new volcanic edifice and related lava field to detect elevation, areal, and volume variations.

Summary of the main findings:

- Subaerial deposit of lava flows and proximal fallout: volume 217.4 ± 6.6 Mm^3^ (voids in the lava field and submerged portion of the two deltas are not considered), subaerial deposition rate 29.5 m^3^/s. In a previous survey carried out on 27/9, 35.8 ± 3.0 Mm^3^ and 59.2 m^3^/s were recorded^26^. Moreover, considering the volume difference between the 27/09 survey^26^ and the post-event survey, the resulting subaerial deposition rate is 27.2 m^3^/s.
- Volcanic edifice: volume 36.5 ± 0.3 Mm^3^ (8.9 ± 0.2 Mm^3^ on 27/9^26^); surface 0.6 km^2^; major and minor axes of the cone, calculated along the main eruptive fracture, approximately 770 (N 140°) and 660 m, respectively; maximum elevation difference 187 m; maximum height 1071.2 m a.s.l.
- Subaerial lava flows: volume 177.6 ± 5.8 Mm^3^ (including fallout deposit on lava flows); surface 11.8 km^2^ (deltas 0.48 km^2^); maximum and average thickness 65 and 15.2 m, respectively; effusion rate 24.1 m^3^/s (submerged volume of lava deltas is not considered).

The present DSM represents a relevant contribution to both the scientific community and the authorities in charge of the restoration activities management.

## Methods

### UAS survey and DSM generation

**Fig. 1:**
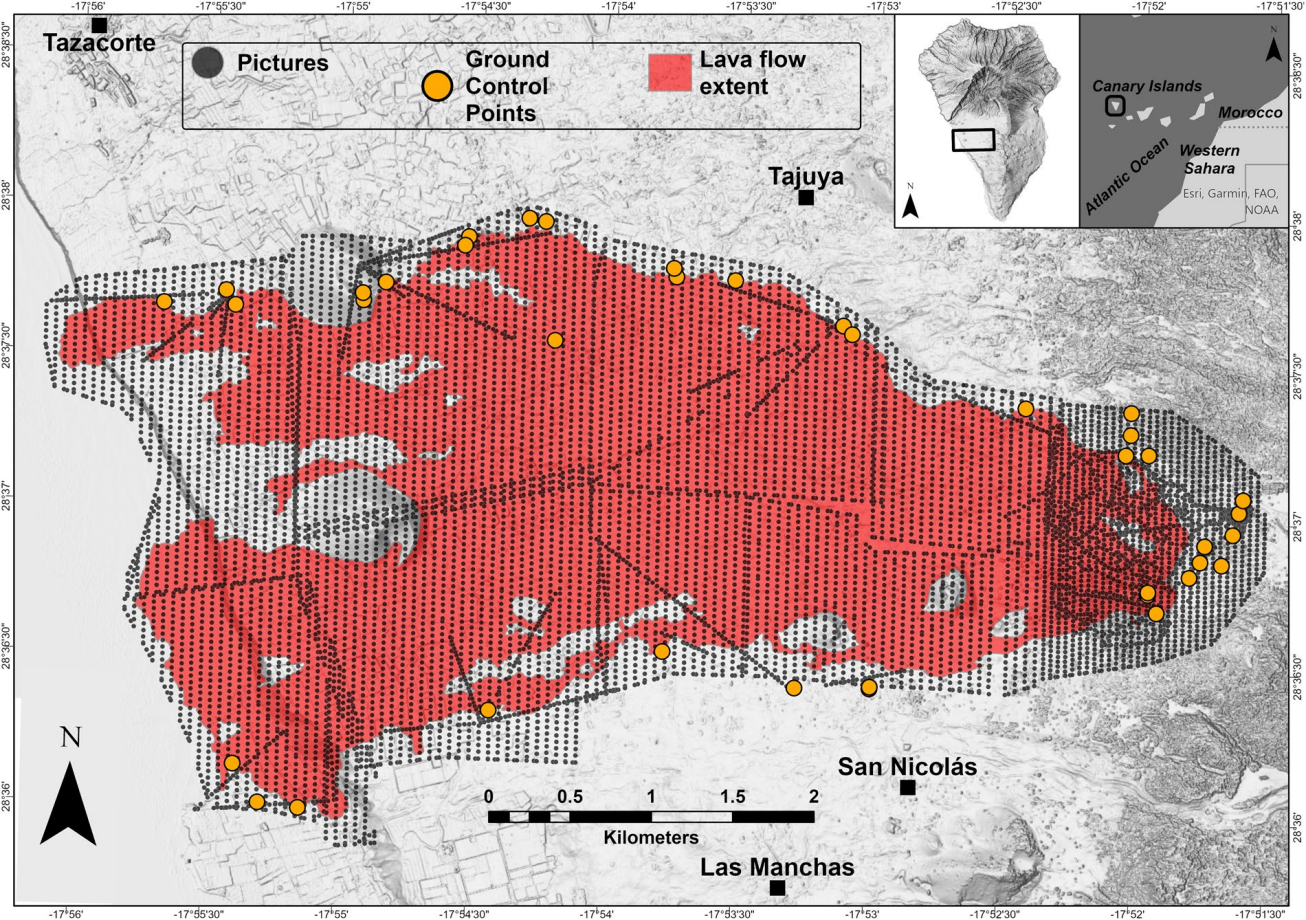
Map identifying the location of each image acquired during the survey (grey dots) and the ground control points used to establish survey control (orange dots). Extent of the lava field (in red) as of 2021-12-18 - [EMSR546] - from Copernicus Emergency Management Service (© 2021 European Union^28^). The inset at the top right of the figure shows the location of La Palma island and the survey area.

We conducted a photographic survey campaign (Fig. 1 and Table 1) between 24 and 28 January 2022, collecting multiple sets of UAS-based high-resolution imagery. We acquired over 12000 aerial pictures using a DJI Phantom 4 RTK UAS with a 1” CMOS 20MP and a field of view (FOV) 84° and 8,8 mm/24 mm (35 mm equivalent) focal length lens.

**Table 1:** Details of the photogrammetric survey data and elaboration.

Number of images	Camera stations	Flying altitude (m a.g.l.)	Ground resolution (cm/pixel)	Tie points	Projections
10,437	9,970	187	4.46	2,751,497	17,509,917
**Reprojection error (pixel)**	**Dense cloud points**	**DSM resolution (cm/pixel)**	**Point density (pts/m**^**2**^**)**	**DSM area (km**^**2**^**)**	
0.642	2,746,820,588	8.91	126	17.16	

A total of 10 multi-flight missions were conducted for the survey, for a cumulative flight path of over 800 km (Fig. 1). All flights except two were nadir image data collection missions, conducted at an approximate altitude of 200 metres above ground level (a.g.l.), resulting in a nominal ground-sampling-distance (GSD) of 5.4 centimetres per-pixel. The 24 and 28 January 2022 flights carried out in the area of the cone were both nadir and oblique image data collection missions conducted at a variable altitude of 50–200 metres a.g.l. For the nadir flights, we flew the UAS using predefined missions. Flight planning was designed with 80% forward and side overlap at ground level. Before each flight, we adjusted the camera’s digital ISO, aperture, and shutter speed according to ambient light conditions.

With respect to other terrains, several additional difficulties characterised the aerial photographic survey campaign at Cumbre Vieja. The cone area has a highly irregular topography, characterised by notched craters and slopes. In addition, viewing conditions at Cumbre Vieja were still partially limited by the presence of vapour/gas plumes and, at times, by atmospheric haze and clouds.

The data on camera position were collected using GNSS-RTK information embedded in the image metadata by means of a DJI D-RTK 2 Mobile Station. In addition, 40 ground control points (GCPs) were distributed along the outer boundary of the lava flow and in the cone area to establish survey control (Fig. 1). In detail, 33 points were used as proper GCPs (i.e., used to georeference and scale the photogrammetric model and for camera calibration purposes), whereas 7 points were used as checkpoints (i.e., not directly used in the photogrammetric modelling process but available to check the accuracy of the generated model). GCPs were measured with a GNSS survey using a DJI D-RTK 2 Mobile Station in real-time kinematic (RTK) mode, with differential corrections sent in real-time by the Instituto Geográfico Nacional differential positioning service available at https://www.ign.es/web/ign/portal/gds-gnss-tiempo-real. The surveyed GCPs have an accuracy of 1–2 cm in horizontal coordinates and 2–4 cm in elevation.

Following image collection, we culled the photoset, removing dark and/or blurry photos. We then processed 9970 georeferenced images using the Agisoft Metashape® software package (version 1.6.3) based on the Structure-from-Motion and multi-view stereo photogrammetry algorithm (SfM–MVS)^12^. The workflow of our photogrammetric analysis included the following: (1) image masking for areas with strong degassing and/or unnecessary background; (2) camera triangulation with image position and orientation and generation of sparse point cloud; (3) filtering of the sparse point cloud to remove points with bad geometry, large pixel matching errors, and large pixel residual errors; (4) generation of the dense point cloud; (5) cleaning of the dense point cloud by using the “filter by confidence” tool and by manually removing anomalous floating points caused by the presence of the volcanic plume; and (6) generation of DSMs and orthomosaics. We set the processing parameters in Agisoft Metashape® to “high” for photo alignment accuracy and “high” quality and “aggressive” depth filtering for dense point cloud generation. For the details of the photogrammetric survey data and elaboration refer to Table 1.

**Fig. 2:**
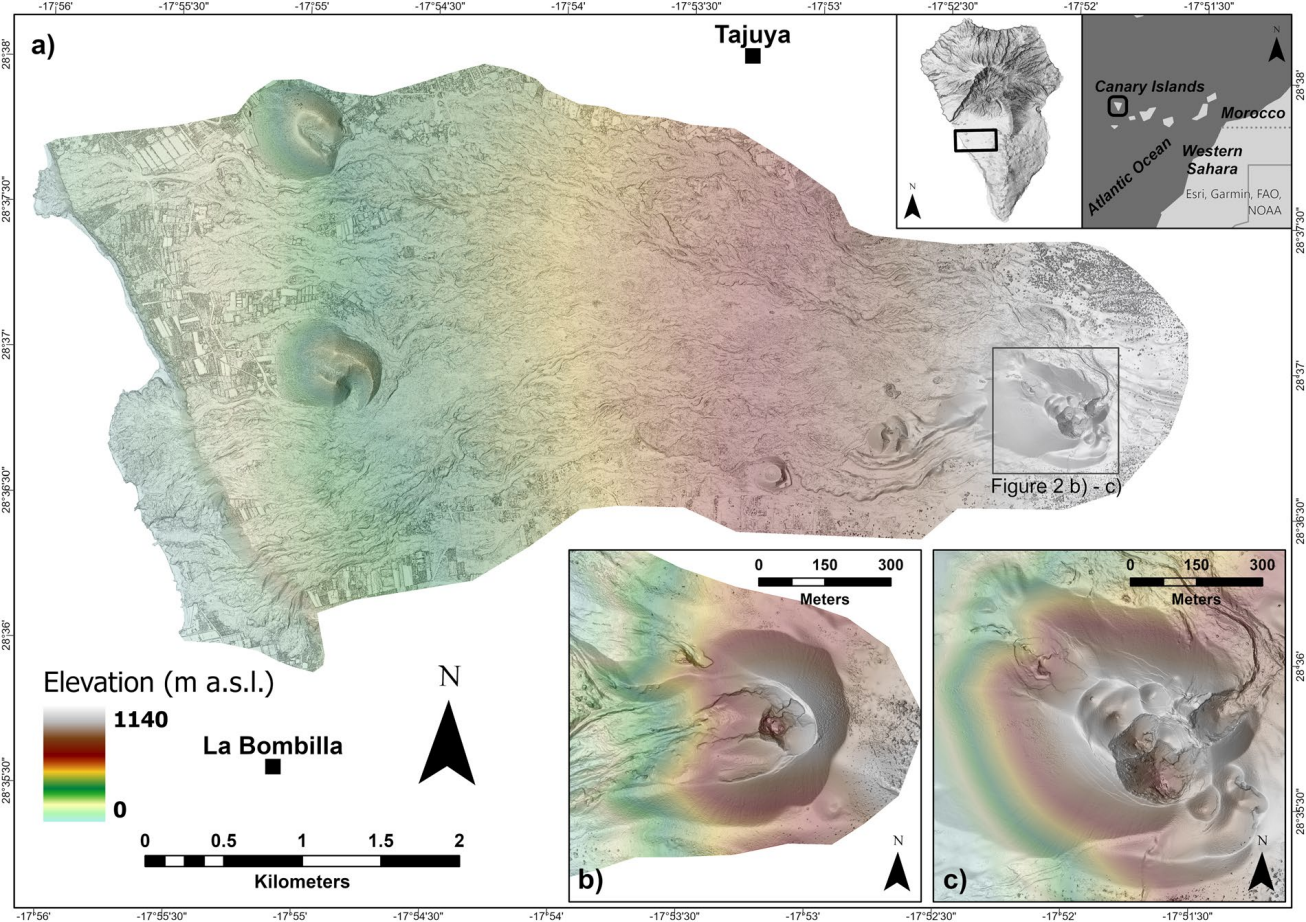
Digital Surface Model (DSM) of the 2021 eruption deposit of Cumbre Vieja volcano. (**a**) Multidirectional hillshade of the DSM. The inset at the top right of the figure shows the location of La Palma island and the survey area. The grey square in the eastern portion of the study area marks the extent of Fig. 2b,c; (**b**) detailed view of the cone on 27 September 2021^26^ and (**c**) in January 2022, respectively.

We generated a 0.2 m/pixel DSM (Fig. 2) and a 0.1 m/pixel orthophotomosaic, covering an area of about 17 km^2^. Unlike a digital elevation model (DEM), the DSM represents the elevation of the highest object within the bounds of a cell. Vegetation, buildings, and other objects have not been removed from the data.

The dataset was processed and analysed in the REGCAN95/UTM zone 28 N [EPSG:4083] Coordinate System. The transformation from ellipsoidal to orthometric heights has been performed using the Geoid model EGM08-REDNAP (https://datos-geodesia.ign.es/geoide/).

### Elevation change detection

**Fig. 3:**
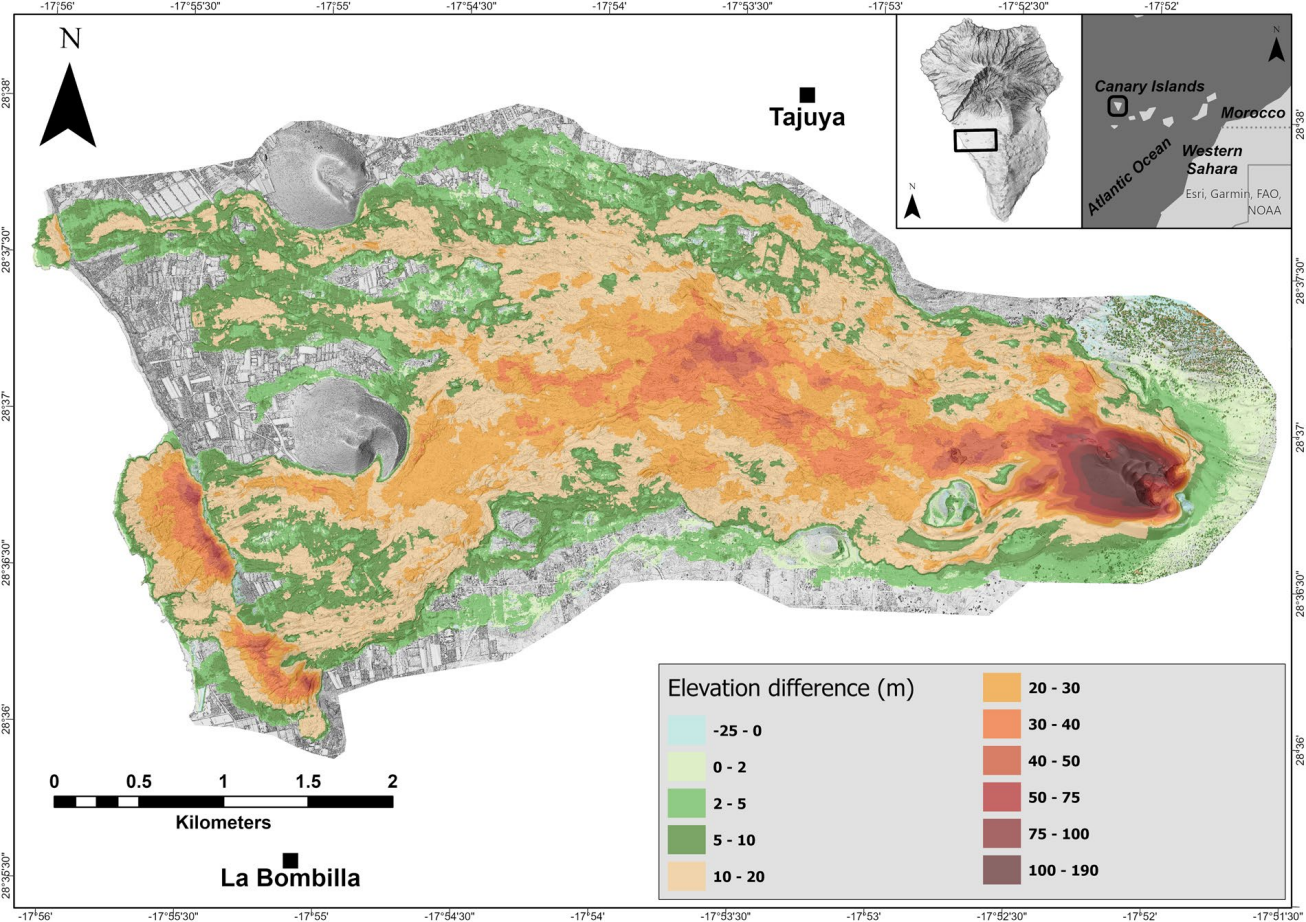
Elevation difference map for the period 2015 - January 2022 (pre- and post-2021 eruption).

Elevation change detection (Fig. 3) was obtained by differencing our surveys and a pre-event 2m-pixel DTM acquired in 2015 for the Spanish PNOA-LiDAR project^25^. The assumption is that between the acquisition of the pre-event DTM (2015) and the beginning of the volcanic activity (19 September 2021), no significant height variation took place in the study area, so that elevation differences obtained in our analysis are mainly linked to the volcanic eruption. To subtract the post- and pre-event surveys, we resampled our DSM to 2 m/pixel resolution (same resolution as the 2015 DTM). Considering the vertical Root Mean Square Error (RMSE) of 0.26 m for our model (before resampling), we set the threshold elevation change (minimum level of detection or minimum elevation change that can confidently be considered a true change) to 0.5 m. It is worth mentioning that the pre-event reference surface is a DTM while our product is a DSM. Such difference must be considered when subtracting both layers as height contributions from vegetation and buildings are still present on the DSM. However, the contribution of such areas is negligible as they are not present above the lava flows and in the cone area.

## Data Records

The data record consists of a high-resolution (0.2 m/pixel) photogrammetric Digital Surface Model processed from survey campaign photographs using Agisoft Metashape®. Details of the photogrammetric survey data and elaboration are summarised in Table 1. The Digital Surface Model was processed and analysed in the REGCAN95/UTM zone 28 N [EPSG:4083] Coordinate System. The dataset is stored in GeoTIFF file format in the OpenTopography repository^27^ and is shared under the CC BY 4.0 use license.

## Technical Validation

Errors in our photogrammetrically-generated DSM result from a complex interplay of geometric and physical parameters, such as image scale, GSD, camera network geometry (nadiral, cross, oblique strips), percentages of image overlap (forward and sidelap), camera shutter speed and exposure settings, lens specifications, image sharpness, camera calibrations, flight design (e.g., flight-line geometry and altitude), surface texture and albedo, lighting conditions, accuracy and distribution of GCPs, disturbances from volcanic activity, as well as on processing: SfM, BBA, image matching, point cloud noise, and outlier removal algorithms.

We therefore applied several strategies to mitigate errors, among which the most important were the following: (1) the use of fast (>1/400 s) camera shutter speeds (i.e., exposure times) whenever possible, (2) the variation of flight altitudes and camera orientation, (3) the application of best practices for processing in Agisoft Metashape, (e.g.^12^), and (4) the removal of sparse cloud points with large uncertainty via Metashape’s gradual selection tools.

The technical quality of the reconstructed DSM was assessed by using the survey report generated by Agisoft Metashape® and by comparing our DSM to a pre-event DTM^25^. According to the Agisoft Metashape® survey report the GCPs and check points error estimates are as follows: the total GCPs Root Mean Square Error (RMSE) is 6.18 cm and the total check points RMSE is 14.59 cm (Table 2). The residual elevation difference with respect to a 2 m/pixel pre-event (2015) DTM^25^ extracted at 13 check points placed in the unchanged regions of our DSM was used as an additional indication of the vertical RMSE, which is 0.26 m. Our model is thus sufficiently accurate for the scale of changes reported in this study.

**Table 2:** Ground control points (GCPs) and check points Root Mean Square Error (RMSE).

Type	Count	X error (cm)	Y error (cm)	Z error (cm)	XY error (cm)	Total (cm)
Ground control points	33	2.22233	2.93895	4.9619	3.68459	6.18035
Check points	7	8.40241	7.77961	9.0432	11.4509	14.5912

X - Easting, Y - Northing, Z - Altitude.

## Data Availability

No custom code was used to generate or process the data described in the manuscript.
